# Discovery of N-glycan Biomarkers for the Canine Osteoarthritis

**DOI:** 10.3390/life10090199

**Published:** 2020-09-14

**Authors:** Hyunjun Lee, Ahyun Lee, Nari Seo, Jiwon Oh, Oh-Kyeong Kweon, Hyun Joo An, Jaehan Kim

**Affiliations:** 1 Department of Food and Nutrition, Chungnam National University, 99 Daehak-ro, Yuseong-gu, Daejeon 34134, Korea; hjlee@o.cnu.ac.kr (H.L.); lah6427@gmail.com (A.L.); 2 Graduate School of Analytical Science and Technology, Chungnam National University, 99 Daehak-ro, Yuseong-gu, Daejeon 34134, Korea; snr85@cnu.ac.kr (N.S.); hjan@cnu.ac.kr (H.J.A.); 3 Department of Veterinary Surgery, College of Veterinary Medicine, Seoul National University, 1 Gwanak-ro, Gwanak-gu, Seoul 08826, Korea; jiwon3903@snu.ac.kr (J.O.); ohkweon@snu.ac.kr (O.-K.K.)

**Keywords:** N-glycan, serum, canine, osteoarthritis, mass spectrometry, biomarker, in vitro diagnosis

## Abstract

Protein glycosylation is a post-translational modification that impacts on protein activity, stability, and interactions. It was sensitively altered by the cellular state and, therefore, is now used for a diagnostic or prognostic indicator of various human diseases such as cancer. To evaluate the clinical feasibility in the veterinary area, the N-glycan biomarkers were discovered from canine serum for the diagnosis of osteoarthritis (OA), which is one of the most common diseases of dogs. N-glycome was obtained from 20 μL of canine serum by the enzymatic cleavage followed by the purification and enrichment using solid-phase extraction. Independent compositions of 163 and 463 N-glycans were found from healthy control (n = 41) and osteoarthritis patients (n = 92), respectively. Initially, 31 of the potential biomarkers were screened by the *p*-values below 1.0 × 10^−10^ from ANOVA. Then, the area under the curve (AUC) and the intensity ratio between OA patient and healthy control (P/C ratio) were calculated. Considering the diagnostic efficacy, the AUC bigger than 0.9 and the P/C ratio larger than 3.0 were used to discover 16 N-glycans as diagnostic biomarkers. Particularly, five of the diagnostic biomarkers were AUC above 0.99 and three of N-glycans had AUC 1.0. The results suggest a clear possibility for N-glycan biomarkers to be used as a clinical tool in the veterinary medical area enabling to provide objective and non-invasive diagnostic information.

## 1. Introduction

Glycosylation is a post-translational modification (PTM) of proteins that occurs during the translocation and trafficking of protein through ER and Golgi [1,2]. By the competitive reaction of the series of glycosyltransferase, hexose (Hex), N-acetylhexosamine (HexNAc), fucose (Fuc), and sialic acid (NeuAc: N-acetylneuraminic acid and NeuGc: N-glycolylneuraminic acid) were polymerized without the inherent genetic template [3,4]. Although N-glycans are composed of less than 10 carbohydrate monomers, their biochemical structure is big, diverse, and complex. As most PTM does, it intervenes in the mode of or the degree of protein functionality that influences the stability and activity of an innate protein, and the recognizability and interaction of ligands [2,5,6,7].

Blood is a collective reservoir that reflects the response of the human body. Consequently, the global profile of serum N-glycome is sensitively changed by the cellular state [8]. With the change of specific protein expression, the profile of N-glycan expression can be affected. Without alteration of protein expression, the change of the cellular environments such as the onset of or the response to the disease readily impacts on the protein glycosylation [9,10].

While the serum N-glycan profile is promising for the blood-based basic and non-invasive in vitro diagnosis, its structural complexity and the diversity hinder the practical application. With the advancement of mass spectrometry, glycomics is now available and becomes crucial to understand the hidden molecular underpinning of the biosystem as well as the disease diagnosis [10,11,12,13]. As an indicator of early diagnosis or prognosis monitoring of disease, N-glycan has been proven for its high efficacy and feasibility. Cancer, in particular, has been intensively studied due to the importance of early diagnosis for treatment and survival [12,14]. A series of N-glycan biomarkers with an area under the curve (AUC) over 0.8 are found from stomach cancer [14,15], pancreatic cancer [16], hepatocellular carcinoma [17], lung cancer [18,19], prostate cancer [20], breast cancer [21], ovarian cancer [22], and colorectal cancer [23]. In addition to cancer, the changes of N-glycan expression in autoimmune diseases, such as rheumatoid arthritis [24] or inflammatory bowel disease (IBD) [25], are studied intensively.

With the increase of the nuclear family, single-person households, late marriage, elderly households, and low birth rates, the number of households living with pets is increasing. At the same time, the meaning of pets is moving from a domestic animal to a family member or a companion for life. Consequently, the veterinary care and clinic market is growing rapidly [26,27] and the demands for disease diagnosis are explosive. However, the biomedical technology that can be utilized in the veterinary clinic has not well fulfilled the demand yet. In particular, the increase of older animals changes the main disease pattern and expands the veterinary diagnostics market reaching USD 3.3 billion globally in 2018 and is expected to grow at a CAGR of 6.4% over the forecast period (2014–2026) [28].

The advance of the clinical methods or the laboratory examinations for the human disease is readily adapted to veterinary medicine, and then it improves the diagnostic methods for pets and cattle. For example, blood tests for the serum biochemicals or immune proteins are widely used [29] and medical instruments such as X-ray or MRI [30,31] have become standard equipment in veterinary clinics. In this prospect, the serum N-glycans can be a valuable biomarker to examine and understand the health condition of animals. Given the fact that animals cannot express their symptoms and conditions verbally, biomarkers that can provide the objective indication of the disease are crucial.

Osteoarthritis (OA) is a common debilitating and degenerative disease [32,33] which occurs in 20% of dogs [34]. While the underlying pathological mechanism of OA is not clearly understood, no early diagnostic methods have been developed to prevent the clinical progress of the disease [35]. The active interventions are available that are used widely to treat other joint diseases such as rheumatoid arthritis; however, only the medical and surgical interventions are provided for remission instead of treatment [36]. Simple radiographs have been used to diagnose arthritis, but early pathological change is not observed making early diagnosis impossible. Even when the bone distortion occurs, often radiographic cannot catch the OA. Besides, radiographic features are generally less relevant to joint function [37]. Due to the inaccuracy of imaging diagnosis and the lack of an appropriate questioning method to patients, the development of biomarkers from synovial fluid, serum, or urine is crucial.

The alteration of glycosylation on the serum proteome has been widely studied and has now become a novel indicator of various diseases in humans [2]. To evaluate the feasibility and the applicability of glycomics in the veterinary clinic area, we discovered the N-glycan biomarkers from canine serum. After enzymatic cleavage from the serum protein, N-glycans were purified and enriched by the solid phase extraction (SPE). Then, the N-glycome of canine serum was analyzed by mass spectrometry. By the quantitative comparison between healthy control and patient groups, potential biomarkers were initially discovered. Then, the applicable biomarkers were selected considering the diagnostic efficacy and analytical stability.

## 2. Materials and Methods

### 2.1. Chemical

Peptide N-glycosidase F (PNGase F) was purchased from New England Biolabs (Ipswich, MA, USA). Solid-phase extraction (SPE) cartridge consisted of porous graphitized carbon (PGC) was obtained from Agilent Technologies (Santa Clara, CA, USA). Acetonitrile (ACN) and other reagents were of HPLC grade.

### 2.2. Dog Serum Samples

The serum samples of 41 healthy control and 92 OA patients were collected by Seoul National University Veterinary Hospital in Korea. All experiments were approved by the Institutional Animal Care and Use Committee of Seoul National University (SNU-180530-3-1). Patients were diagnosed by the combination of lameness, pain response, joint capsule inflammation score, X-ray OA score, and WBC count.

### 2.3. Serum N-glycan Release

In total, 20 μL of canine serum was mixed with 20 μL of 200 mM ammonium bicarbonate buffer containing 10 mM dithiothreitol. The mixed sample was alternately placed in boiling water for 10 s and cooling water for 10 s. The procedure was repeated for 2 min to denature the proteins. 500 units of PNGase F was added to the sample after cooling enough. The mixture was incubated at 37 °C for 16 h in a water bath. 200 μL of chilled ethanol was added and then kept in the −80 °C freezer for 40 min. Protein precipitation was removed after centrifugation. The supernatant was collected in a 1.5 mL new tube and then dried in a rotary evaporator.

### 2.4. Purification and Enrichment

Released N-glycans were purified and fractionated by PGC-SPE. The dried sample was dissolved in 500 μL of water and kept at 4 °C for 1 h. Briefly, the cartridge was activated by 6 mL of water and washed by 6 mL of 80% ACN with 0.1% TFA. After preconditioning with 6 mL of water, the dissolved sample was loaded into the cartridge. The sample tube and tip were rinsed by 500 μL of water and re-loaded into the cartridge. Excessive detergents and salts were removed by 6 mL of water. The N-glycans were eluted with 10% ACN, 20% ACN, and 40% ACN with 0.05% TFA, sequentially. Eluted fractions were vacuum-dried before LC/MS analysis. 

### 2.5. Analysis of N-glycan by Nano-LC Chip Q-TOF MS and MS/MS

Each SPE fraction was reconstituted by 15 μL of water. Glycan samples were analyzed by Agilent 6540 Nano-LC chip Q-TOF MS system. N-glycans were separated using a graphitized carbon-based chip column composed of 40 nL enrichment column and 75 μm × 43 mm separation column (Agilent Technologies, Santa Clara, CA, USA). The gradient was applied to the nano pump using (A) 3% ACN with 0.1% FA and (B) 90% ACN with 0.1% FA with 0.4 μL/min flow rate as follows: 0–2.5 min, 3% B; 2.5–20 min, 3–16% B; 20–30 min, 16–44% B; 30–35 min, 44–100% B; 35–45 min, 100% B; 45–45.1 min; 100–3% B, maintained 3% B for 19.9 min to equilibrate the column. The capillary pump was operated to deliver the sample to the enrichment column with 4 μL/min flow rate of 3% solvent A. The Q-TOF MS system was performed in the positive ion detection mode for MS scans and auto MS/MS analysis. The *m/z* 500–2500 and *m/z* 100–3000 of the mass range were applied for MS and MS/MS, respectively. Acquisition rates were 2.0 s/spectra for MS and 0.63 s/spectra for MS/MS. The drying gas temperature was 325 °C and the gas flow was 5 L/min. 

### 2.6. Data Processing and Statistical Analysis

Raw LC-MS data were processed by the MassHunter Qualitative Analysis software (version B.7.00 SP1, Agilent Technologies). All MS peaks of ion species were filtered based on expected isotopic distribution with a signal-to-noise ratio of 5.0. The possible glycan composition was extracted based on the in-house N-glycan library using the Molecular Feature Extraction (MFE) function equipped on MassHunter software, initially. Then, the compositions were assigned by accurate mass (10 < ppm), isotopic pattern, and glycan correlation. After composition assignment, the major N-glycan structure was deduced from the MS/MS and the comparison to the in-house library. The structure of minor glycan was tentatively assigned by the N-glycan synthesis pathway and their correlation [38,39]. 

N-glycan was named using the 5-digit annotation method. Five numbers connected by an underbar indicated the number of hexose (Hex), N-acetylhexosamine (HexNAc), fucose (Fuc), N-acetylneuraminic acid (NeuAc), and N-glycolylneuraminic acid (NeuGc), respectively. For example, 4_4_2_1_0 means the N-glycan was composed of 4 of Hex, 4 of HexNAc, 2 of Fuc, 1 of NeuAc, and no NeuGc. The search was performed within the mass error of 10 ppm. Biomarkers were selected by the comparison of patients and healthy controls using the normalized absolute peak intensity (*NAPI*) calculated as follows [22,40];

(1) $$NAPI=\frac{absolute\;peak\;intensity\;\left(API\right)}{sum\;of\;total\;API}\times 1000$$

With the *NAPI*, the accumulated intensity of each N-glycan was calculated additionally. When the *NAPI* of each N-glycan was ranked from highest to lowest, the accumulated intensity of specific N-glycan is the percentile of the sum of the NAPIs that had the higher ranks.

To discover the biomarkers, initially, monoisotopic peaks with the value smaller than 10^−10^ from ANOVA were selected as potential biomarkers. Then, the receiver operating characteristic (ROC) curve of each potential biomarker was calculated to decide the classification efficiency of biomarkers. Eventually, the peaks with the area under the curve (AUC) of the ROC curve greater than 0.9 were chosen as the biomarker.

## 3. Results and Discussion

### 3.1. MS Data Analysis for Serum N-glycan Annotation

The number of N-glycans in the canine serum was summarized in Table 1. Initially, 140~160 of N-glycans extracted by the MFE software from each serum sample in the healthy control group and 180~240 of N-glycans from the osteoarthritis (OA) patient group. After the integration of each independent *m/z* and the corresponding intensity data into a single list of *m/z*, 317 and 598 compositions of N-glycans were found in the healthy control and patient group, respectively. 

To avoid false annotation, data were filtrated using the frequency parameter (*f*) of each *m/z*. The N-glycan compositions that observed less than 10% of samples (*f* < 10%) in a group were ignored as noise. As a result, the number of N-glycans was reduced to 163 in a healthy control group and 463 in an OA patient group. Among the initial annotation, 49% of N-glycans in the healthy control group were observed in less than 10% of samples and subsequently eliminated as noise (Supplementary Table S1). In OA patient groups, meanwhile, only 23% of initial annotations fell into the same category. However, 28% of initial annotations in the OA patient group were still exhibited as only 10~20% of samples. Given the fact that the sample size of the OA patient group was twice the healthy control group, the number of N-glycans that can be ignored, seemed similar between the two groups. Although the OA patient group exhibited more diverse N-glycan profiles than the healthy patient group, the percentile of housekeeping N-glycans that exhibited more than 90% of samples was close at 17% and 11% (Supplementary Table S1). 

After the data filtration, the N-glycans observed in a sample group were classified into three categories based on their intensity parameter which represents the amount of each N-glycan. When the N-glycans were ranked by their intensity from the highest to lowest, the groups of N-glycans accounted for 95% of total intensity were considered the “Major class” and 95~99% of total intensity were the “Minor class”. Then, the remaining N-glycans that composed 99~100% of total intensity were designated “Trace”. As shown in Table 1, only 36 and 50 of N-glycans were occupied at 95% of total intensity (Major class), which accounts for 22.1% and 10.8% of the total assigned N-glycans in the healthy control and OA patient groups, respectively. Another 20% of N-glycans were classified into the “Minor class” and the remaining 60~67% of N-glycans were in the “Trace” group. The peak intensity distribution of both healthy control and OA patient groups was similar to each other (Supplementary Figure S1). The peak intensities of N-glycans in the “Major class” were about 20 folds higher than those in the “Minor class”. N-glycans in the “Trace” group had a peak intensity of less than 2, which was also 20 folds smaller than the Minor class.

This frequency-based filtration brought the deviation of an overall number of N-glycan assignments. While 162 of N-glycans were assigned on the control group, 463 of N-glycans were assigned to the patient group. However, the number of major N-glycans that occupied 95% of the total N-glycans was close to 36 and 50 in the control and patient group, respectively. Further, 35 of them were shown in both groups. Major deviation of the N-glycan number was observed in the “Minor” and particularly the “Trace” class. While Major class N-glycans have the frequency over 90%, the frequency of Trace class N-glycans was less than 40%. Among 311 of Trace class N-glycans in the Patient group, 305 of N-glycans exhibited the frequency less than 40% and, moreover, 244 of N-glycans had a frequency less than 20% (Supplementary Table S2). It suggested that the deviation of the number of total assigned N-glycans between two groups occurred due to the difference of sample numbers and the frequency-based filtering as well as the characteristics of two sample groups. The list of N-glycans in Major and Minor class is listed in Supplementary Table S3.

### 3.2. Global Comparison of N-glycan Profile

**Figure 1:**
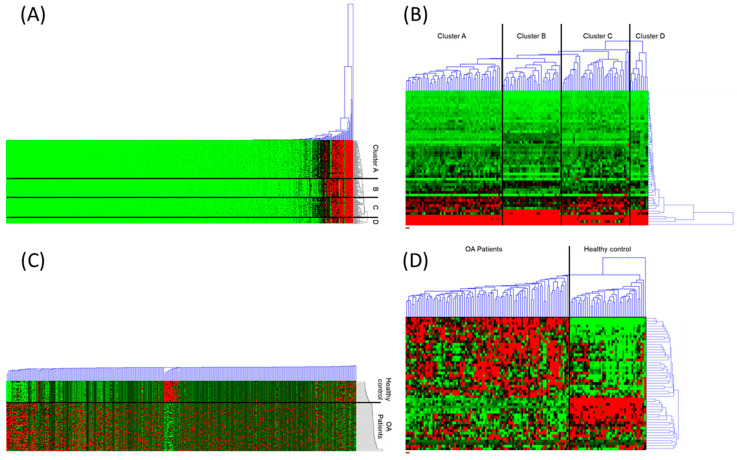
The hierarchical cluster analyses of N-glycans were assigned. (**A**) the total N-glycan, (**B**) the major N-glycan only, (**C**) total N-glycans after the standardization by z-score, (**D**) the major N-glycans after standardized by z-score.

The expression profiles of the serum N-glycans were compared. The hierarchical cluster analyses with the heatmaps were described in Figure 1. The Euclidean distance has been used to calculate the similarity matrix between samples. The cluster was constructed by the average linkage method based on the similarity index. As shown in Figure 1A, samples were clustered into four groups, two for the healthy control (clusters B and D) and another two for the OA patients group (clusters A and C). No common characteristics were found between the samples within the same cluster. However, the distributions of the similarity distance in each of the four clusters were similar to the range between 0.77 and 0.97. When the analysis was performed using major glycans only, clustering results were almost identical except two of the OA patient samples, which were joined into the cluster group D, which was the control sample cluster (Figure 1B). 

The remarkable changes have been observed when the N-glycan intensity was standardized by the z-score using the mean and the standard deviation of each N-glycan (Figure 1C). Samples gathered into two clusters showing a very distinct pattern of N-glycan expression. Due to the were standardization, the difference between minor glycans was clearly exhibited and influenced the clustering result (Figure 1A,C). When the major glycans have been used for the hierarchical cluster analysis, the pattern of standardized N-glycan expression was divided into two clusters that exclusively contained the healthy control and OA patients.

### 3.3. Discovery of Potential N-glycan Biomarkers

For the initial screening of the potential OA biomarkers, the absence of an intensity value needs to be addressed whether missing data or an intensity value of zero. Mass spectrometric analysis is the positive detection method that the analytical results can be discussed on only when the signal is obtained. When the mass spectrometry cannot acquire the intensity value of a specific *m/z*, there always could be a possibility that the target molecule is not detected in those specific analytical conditions (missing values), or no such molecules are present in the sample (value of zero). In the meantime, the diagnostic decision has to be made whether the signal is obtained or not, and, consequently, biomarkers for the diagnosis of disease should address those non-observational data issues.

To discover the intensity-based biomarkers, no observational data (blank cell in data process) were filled by the value of zero in two conditions. When the major glycans have been used the OA patient group should have a frequency above 95%. It suggested that, for the diagnostic purpose, a marker should observe at least one group of the sample clearly. Second, the intensity of the low-frequency group that contains many blank cells without observational data, has to be smaller than the other high frequency (>95%) group. For example, an N-glycan of 4_4_3_0_0 (4Hex 4HexNAc 3Fuc 0NeuAc 0NeuGc) was observed from all the samples in the OA patient group (*f* = 100%) and the average peak intensity was 35.0 ± 11.7. In the meantime, the same N-glycan has been observed only 16 times among 41 samples (*f* = 39%) of healthy control samples when the average peak intensity of non-blank cells was 3.1 ± 2.9. In this case, the intensity value of zero was filled into the blank cell of the OA patient group with the consideration that no observation was caused by the low concentration of the molecules under the detection limit of the machine. Consequently, the sample number of the OA patient became 41 instead of 16 and the average came to be 1.2 ± 2.3. 

After addressing the blank cell issue, ANOVA analysis has been performed to discover the potential OA biomarkers. The N-glycans only with the *p*-value < 10^−11^ were selected as a potential biomarker (Table 2). A total of 31 N-glycans were found to be potential markers that exhibited the *p*-value from 2.7 × 10^−66^ to 8.7 × 10^−11^. 

### 3.4. Efficacy of N-glycan Biomarkers

The efficacy of biomarkers was not determined by the *p*-value from ANOVA. Two parameters were chosen to evaluate the diagnostic power of potential biomarkers. First, the Area Under the Curve (AUC) of the Receiver Operating Characteristics (ROC) curve of each potential marker should be larger than 0.9. Among 31 potential biomarkers that had the *p*-value < 10^−11^, 25 of N-glycans exhibited the AUC larger than 0.9 (Table 3). 

Second, the P/C ratio was considered due to the practical reason for the diagnosis. The P/C ratio is the ratio between the average intensity of the OA patient samples (P) and the control samples (C). Despite the inherent advantages such as high sensitivity and accuracy, the mass spectrometry exhibited an unstable quantitative reproducibility depending on the samples and experimental conditions. During the repetitive measurement of a sample, the relative standard deviation (RSD) of the peak intensity was in the range between 5% and up to 25%. Particularly, the minor glycans that have a small peak intensity tend to have large RSD. In the meantime, biomarkers were developed by the statistical analysis of the numerical data obtained from the discovery set. Regardless of biological meaning or molecular characteristics, biomarkers were listed by the statistical comparison and segregation of the numbers between two groups of samples. In other words, the standard deviation which represents the distribution of numbers became one of the most important to be discovered as potential biomarkers. For example, biomarkers that have the P/C ratio close to +1 or −1, even if the biomarker has a high AUC, could be discovered because of a small RSD rather than the significant alteration of N-glycan expression level (Compare the Supplementary Figure S2A–C). If the P/C ratio was close to ±1, the diagnosis result could be changed by the small variation of instrumental measurement or human error.

To overcome the constraint of the reproducibility and the subsequent diagnostic stability, a P/C ratio over ±3.0 was separately designated as diagnostic biomarkers (Table 3A). Including five major N-glycans, sixteen of the N-glycans were found to be the diagnostic biomarkers. The remaining eight of potential N-glycans that have the AUC > 0.9 but the P/C ratio < ±3.0 were categorized as the auxiliary biomarkers (Table 3B). 

**Figure 2:**
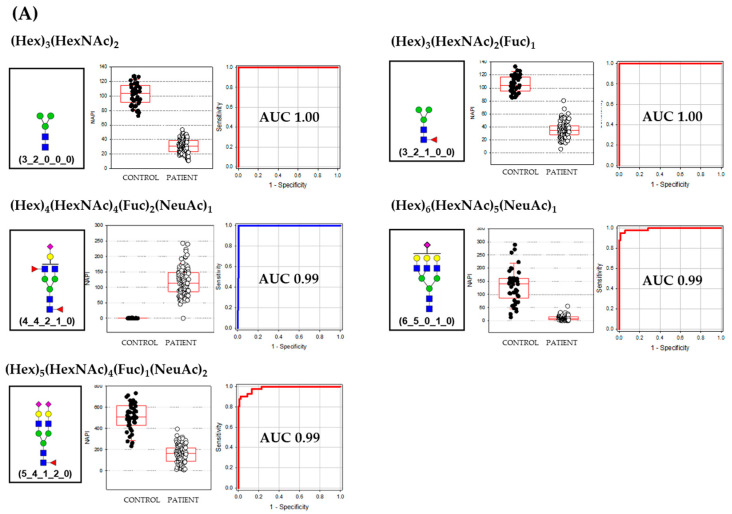

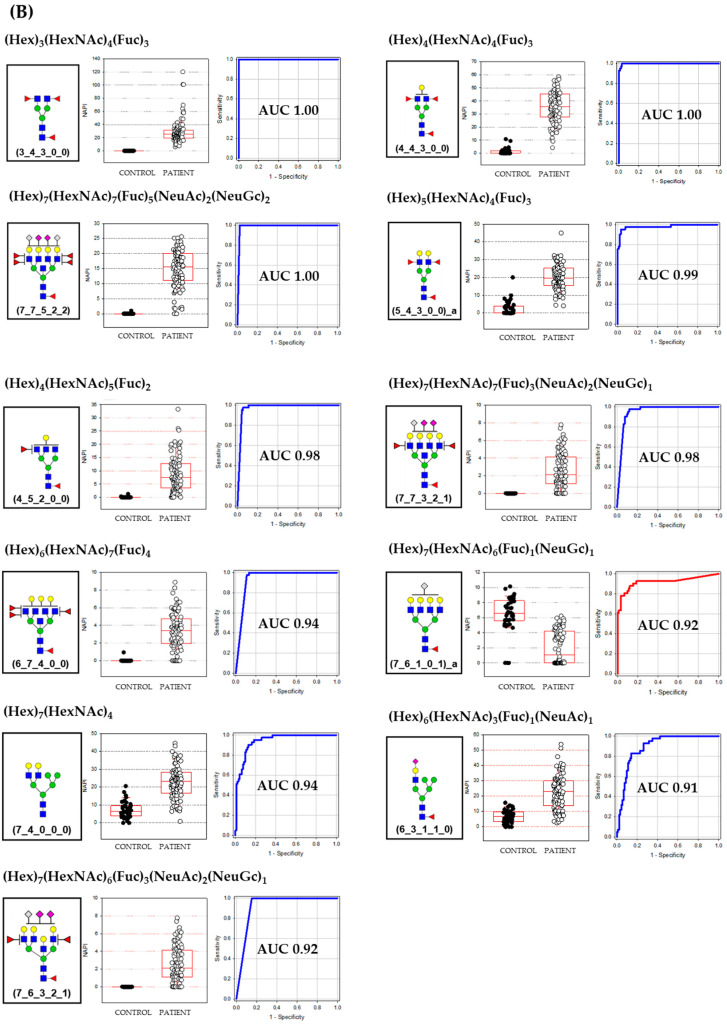
The Diagnostic biomarkers; tentative N-glycan structures, boxplot of healthy control and patient group samples, and receiver operating characteristic (ROC) curve. Redline in the ROC curve indicates the average intensity of healthy control group is higher than OA patient group (**A**) major class glycans, (**B**) minor class glycans.

The AUCs of a major class of biomarkers were over 0.99 and the sizes of the N-glycan molecules were relatively smaller than minor class biomarkers. Major class biomarkers were core and a complex type such as bi-antennary and tri-antennary N-glycans (Figure 2A). The major class glycans were more highly expressed in the healthy control group except for the difucosyl-monosialylated bi-antennary glycan (4_4_2_1_0). Meanwhile, most of the minor class glycan biomarkers were complex type N-glycans and exhibited the broad spectrum of their size from bi-antennary to bisecting tetra-antennary structures (Figure 2B). Their AUCs were from 1.0 to 0.91 and, interestingly, were highly expressed in OA patients group except for one tetra-antennary N-glycans decorated by one fucose and one N-glycolylneuraminic acid (7_6_1_0_1). The average size of the minor class glycan biomarker was the degree of polymerization (DP) 14.5 ± 4.5 which is 5 units bigger than the major class glycan biomarker (DP 9.2 ± 3.4). Another unique characteristic that differs between major and minor class biomarkers was the decoration by fucose and sialic acid. While the degree of decoration in major class biomarkers was DP 1.6 ± 1.3, the minor class glycan biomarker was highly decorated showing the DP of 3.6 ± 2.5 units. The average composition of OA biomarkers was (Hex)_5.25_ (HexNAc)_4.63_ (Fuc)_2.00_ (NeuAc)_0.69_ (NeuGc)_0.31_ showing a highly decorated structure by two fucose and one sialic acid (0.69 NeuAc + 0.31 NeuGc).

## 4. Conclusions

We have discovered biomarkers from the serum N-glycome to diagnose canine osteoarthritis. Using mass spectrometry as a diagnostic instrument, the feasibility of an N-glycan biomarker system in the clinical area of veterinary medicine has been studied. Sixteen N-glycans were discovered that exhibited AUCs more than 0.9 to 1.0 with excellent diagnostic stability. Although a wide range of validation has to be followed, this study suggests the strong potentials that the alteration of serum N-glycans expression could be an emerging indicator of in vitro diagnostics (IVD) in veterinary clinics.

## Figures and Tables

**Table 1:** The number of N-glycans in canine serum.

		Healthy Control	OA Patient	Shared ^a^
Number of Samples		41	92	
Number of N-glycan compositions	Extracted	317	598	
	Filtrated (*f* > 10%) ^b^	163	463	160
	Major class (Acc. Int. ≤ 95%) ^c^	36 (22.1%)	50 (10.8%)	35
	Minor class (Acc. Int. 95~99%)	30 (18.4%)	102 (22.0%)	18
	Trace (Acc. Int. ≥ 9 9%)	97 (59.5%)	311 (67.2%)	45

^a^ Number of N-glycan compositions presented both healthy control and patient samples. ^b^ Frequency parameter, the percentile of samples that have the N-glycan. ^c^ Accumulated intensity.

**Table 2:** The list of the potential osteoarthritis (OA) biomarkers.

*m/z*	N-glycan ^a^		Healthy Control					OA Patient				*p*-Value
		*f* ^b^	AVE ± SD	CV	Rank	Acc. ^c^	*f* ^b^	AVE ± SD	CV	Rank	Acc. ^c^	
910.3277	3_2_0_0_0	100%	102.3 ± 14.9	14.6%	22	84.1%	100%	33.8 ± 8.5	25.1%	45	93.2%	2.7 × 10^−66^
1056.3856	3_2_1_0_0	100%	106.6 ± 13.6	12.7%	17	78.9%	100%	34.7 ± 12.4	35.7%	44	92.8%	1.4 × 10^−60^
1754.6602	3_4_3_0_0	0%	0.0 ± 0.0		354	100.0%	100%	24.3 ± 7.9	32.6%	49	94.2%	1.0 × 10^−40^
1916.7130	4_4_3_0_0	39%	1.2 ± 2.3	191.9%	95	99.6%	100%	35.0 ± 11.7	33.5%	43	92.5%	8.0 × 10^−38^
2368.8408	5_4_1_2_0	100%	507.0 ± 130.8	25.8%	6	60.6%	100%	160.0 ± 86.1	53.8%	14	70.4%	1.5 × 10^−37^
2296.8197	6_5_0_1_0	100%	131.9 ± 64.7	49.0%	14	75.6%	88%	9.4 ± 8.7	92.1%	64	96.4%	5.5 × 10^−37^
2061.7505	4_4_2_1_0	22%	0.3 ± 0.5	208.6%	149	99.8%	99%	118.9 ± 45.6	38.4%	17	74.2%	4.7 × 10^−34^
4500.5968	7_7_5_2_2	2%	0.0 ± 0.2	640.3%	299	100.0%	98%	14.9 ± 6.3	42.0%	56	95.5%	1.1 × 10^−30^
1566.5553	4_3_0_1_0	100%	218.8 ± 37.8	17.3%	8	65.2%	100%	115.1 ± 36.1	31.3%	19	76.5%	2.0 × 10^−30^
2078.7658	5_4_3_0_0	46%	2.5 ± 4.0	159.8%	79	99.3%	100%	20.3 ± 7.2	35.3%	53	95.0%	5.1 × 10^−30^
2239.7982	6_4_1_1_0	100%	81.3 ± 35.9	44.1%	29	90.2%	100%	217.1 ± 56.0	25.8%	11	65.7%	2.0 × 10^−28^
1275.4599	4_3_0_0_0	100%	105.6 ± 18.3	17.4%	18	79.9%	100%	60.7 ± 17.6	29.0%	31	87.1%	2.4 × 10^−26^
1712.6132	4_3_1_1_0	100%	25.6 ± 9.0	35.3%	40	95.7%	97%	11.4 ± 5.6	49.6%	60	96.0%	2.0 × 10^−20^
1964.6977	7_4_0_0_0	95%	7.1 ± 4.5	63.6%	63	98.6%	100%	22.8 ± 8.8	38.8%	50	94.4%	1.2 × 10^−19^
2824.0047	7_6_1_0_1	93%	6.5 ± 2.3	36.1%	66	98.8%	55%	2.1 ± 2.2	105.8%	108	98.1%	4.3 × 10^−19^
2996.1147	6_7_4_0_0	2%	0.0 ± 0.1	640.3%	303	100.0%	89%	3.3 ± 2.1	62.4%	86	97.5%	3.1 × 10^−18^
1259.4650	3_3_1_0_0	100%	559.1 ± 72.7	13.0%	5	55.6%	100%	374.7 ± 111.6	29.8%	5	49.0%	4.4 × 10^−17^
2093.7403	6_4_0_1_0	100%	198.5 ± 57.3	28.9%	9	67.2%	100%	321.4 ± 71.9	22.4%	7	55.6%	5.7 × 10^−17^
1437.5127	5_3_0_0_0	100%	27.4 ± 9.4	34.2%	39	95.4%	100%	53.3 ± 16.7	31.4%	35	89.3%	5.3 × 10^−16^
2077.7454	5_4_1_1_0	100%	198.3 ± 63.2	31.9%	10	69.1%	100%	356.6 ± 103.0	28.9%	6	52.4%	1.3 × 10^−15^
1582.5502	4_3_0_0_1	100%	14.9 ± 7.3	49.1%	49	97.3%	95%	6.1 ± 3.9	63.9%	72	97.0%	2.3 × 10^−15^
1599.5655	6_3_0_0_0	100%	8.3 ± 3.6	43.1%	57	98.2%	100%	19.3 ± 7.5	38.7%	54	95.2%	2.6 × 10^−15^
3901.3907	7_7_3_2_1	17%	0.3 ± 0.9	271.5%	139	99.8%	93%	11.5 ± 8.1	69.9%	58	95.8%	4.7 × 10^−15^
1745.6234	6_3_1_0_0	100%	12.0 ± 7.9	65.6%	52	97.7%	100%	36.2 ± 16.6	45.9%	42	92.2%	4.8 × 10^−15^
1421.5178	4_3_1_0_0	100%	184.4 ± 45.2	24.5%	12	72.8%	100%	118.0 ± 39.0	33.0%	18	75.4%	1.7 × 10^−14^
2036.7188	6_3_1_1_0	90%	6.7 ± 4.2	62.7%	65	98.8%	100%	22.4 ± 11.3	50.6%	51	94.6%	1.9 × 10^−14^
1462.5444	3_4_1_0_0	100%	1301.9 ± 137.8	10.6%	2	28.6%	100%	984.8 ± 219.8	22.3%	2	28.9%	3.5 × 10^−14^
1973.7345	4_5_2_0_0	5%	0.0 ± 0.2	530.9%	264	100.0%	96%	9.0 ± 6.8	75.8%	65	96.5%	6.7 × 10^−14^
1947.6824	5_4_0_0_1	100%	84.7 ± 36.7	43.3%	26	87.7%	100%	160.8 ± 54.0	33.6%	13	68.9%	1.8 × 10^−13^
2255.7931	7_4_0_1_0	100%	52.0 ± 25.5	49.2%	35	93.9%	100%	110.1 ± 45.9	41.6%	21	78.6%	5.0 × 10^−12^
2109.7352	6_4_0_0_1	98%	17.0 ± 10.9	64.0%	47	97.0%	100%	31.8 ± 11.3	35.4%	46	93.5%	8.7 × 10^−11^

^a^ 5-digit N-glycan annotation; ^b^ Frequency of observation among the sample group. ^c^ Accumulated intensity.

**Table 3:** The list of selected biomarkers for OA patients. (A) Diagnostic biomarkers with the P/C ratio > 3-fold, (B) Auxiliary biomarkers with the P/C ration < 3-fold.

	Category	*m/z*	N-glycan	Class ^a^	AUC	P/C Ratio ^b^	*p*-Value
(**A**)	Major	910.3277	3_2_0_0_0	CORE	1.000	−3.0	2.7 × 10^−66^
		1056.3856	3_2_1_0_0	C/HB-F	1.000	−3.1	1.4 × 10^−60^
		2061.7505	4_4_2_1_0	C-FS	0.993	461.6	4.7 × 10^−34^
		2296.8197	6_5_0_1_0	C-S	0.991	−14.0	5.5 × 10^−37^
		2368.8408	5_4_1_2_0	C-FS	0.985	−3.2	1.5 × 10^−37^
	Minor	1754.6602	3_4_3_0_0	C-F	1.000	A/P	1.0 × 10^−40^
		1916.7130	4_4_3_0_0	C-F	0.998	28.6	8.0 × 10^−38^
		4500.5968	7_7_5_2_2	C-FS	0.989	603.7	1.1 × 10^−30^
		2078.7658	5_4_3_0_0	C/HB-F	0.981	8.2	5.1 × 10^−30^
		1973.7345	4_5_2_0_0	C-F	0.975	234.7	6.7 × 10^−14^
		3901.3907	7_7_3_2_1	C-FS	0.954	36.1	4.7 × 10^−15^
		2996.1147	6_7_4_0_0	C-FS	0.944	146.8	3.1 × 10^−18^
		1964.6977	7_4_0_0_0	HB	0.940	3.2	1.2 × 10^−19^
		3698.3113	7_6_3_2_1	C-FS	0.924	A/P	1.6 × 10^−13^
		2824.0047	7_6_1_0_1	C-FS	0.920	−3.1	4.3 × 10^−19^
		2036.7188	6_3_1_1_0	HB-FS	0.906	3.4	1.9 × 10^−14^
(**B**)	Major	2239.7982	6_4_1_1_0	HB-FS	0.973	2.7	2.0 × 10^−28^
		1566.5553	4_3_0_1_0	C/HB-S	0.969	−1.9	2.0 × 10^−30^
		1275.4599	4_3_0_0_0	C/HB	0.955	−1.7	2.4 × 10^−26^
		1259.4650	3_3_1_0_0	C/HB-F	0.921	−1.5	4.4 × 10^−17^
		1437.5127	5_3_0_0_0	HB	0.921	1.9	5.3 × 10^−16^
		2093.7403	6_4_0_1_0	HB-S	0.911	1.6	5.7 × 10^−17^
		2077.7454	5_4_1_1_0	C/HB-FS	0.909	1.8	1.3 × 10^−15^
		1462.5444	3_4_1_0_0	C-F	0.900	−1.3	3.5 × 10^−14^
	Minor	1599.5655	6_3_0_0_0	HB	0.921	2.3	2.6 × 10^−15^

^a^ Type of N-glycans. C: complex type, HB: hybrid type, F: fucosylated, S: sialylated, FS: fucosylated and sialylated N-glycans; ^b^ P/C ratio indicates the ratio between the average intensities of OA patient samples to the healthy control samples.

## Supplementary Materials

The following are available online at https://www.mdpi.com/2075-1729/10/9/199/s1, Figure S1: The comparison of peak intensity between Peak Major class, Minor class, and Trace groups. (A) healthy control group, (B) Osteoarthritis patient group, Figure S2: Example of biomarkers with high AUC but low P/C ratio. (A) and (B) has high p-value and low AUC. (C) has low p-value and high AUC but a similar P/C ratio with (A) and (B). Although N-glycan (C) has a high AUC of 0.90 values of OA patients and healthy control made clusters in close range, Table S1: The percentiles of samples on frequency distribution, Table S2: The number of N-glycans in each class based on the frequency. (A) healthy control and (B) patient group, Table S3: The composition, frequency, and the class of N-glycans assigned (A) healthy control and (B) patient group.
