# Supplementary material for: Discovery of N-glycan Biomarkers for the Canine Osteoarthritis
Source: Life (Basel). 2020 Sep 14;10(9):199. doi: 10.3390/life10090199 (PMC7555374; doi:10.3390/life10090199)
Supplement: Supplementary file 1 [file life-10-00199-s001.pdf]

life

Supplementary Materials

# Discovery of N-glycan Biomarkers for the Canine Osteoarthritis

**Hyunjun Lee [1], Ahyun Lee [1], Nari Seo [2], Jiwon Oh [3], Oh-Kyeong Kweon [3], Hyun Joo An [2] and Jaehan Kim [1,*]**

[1] Department of Food and Nutrition, Chungnam National University, 99 Daehak-ro, Yuseong-gu, Daejeon 34134, Korea; hjlee@o.cnu.ac.kr (H.L.); lah6427@gmail.com (A.L.)
[2] Graduate School of Analytical Science and Technology, Chungnam National University, 99 Daehak-ro, Yuseong-gu, Daejeon 34134, Korea; snr85@cnu.ac.kr (N.S.); hjan@cnu.ac.kr (H.J.A.)
[3] Department of Veterinary Surgery, College of Veterinary Medicine, Seoul National University, 1 Gwanak-ro, Gwanak-gu, Seoul 08826, Korea; jiwon3903@snu.ac.kr (J.O.); ohkweon@snu.ac.kr (O.-K.K.)
* Correspondence: jaykim@cnu.ac.kr; Tel.: +82-42-821-6834; Fax: +82-42-821-8887

Received: 20 August 2020; Accepted: 10 September 2020; Published: 14 September 2020

**Table S1.** The percentiles of samples on frequency distribution.

| *f* | Healthy Control | | Patient | |
|---|---|---|---|---|
| | N-glycan | % | N-glycan | % |
| 100~90% | 55 | 17% | 64 | 11% |
| 90~80% | 4 | 1% | 13 | 2% |
| 80~70% | 5 | 2% | 11 | 2% |
| 70~60% | 6 | 2% | 17 | 3% |
| 60~50% | 10 | 3% | 27 | 5% |
| 50~40% | 11 | 3% | 43 | 7% |
| 40~30% | 12 | 4% | 42 | 7% |
| 30~20% | 20 | 6% | 81 | 14% |
| 20~10% | 40 | 13% | 165 | 28% |
| <10% | 154 | 49% | 135 | 23% |
| Total | 317 | | 598 | |

**Table S2.** The number of N-glycans in each class based on the frequency. (A) healthy control and (B) patient group.

(A)

| *f* | **Total** | **Major** | **Minor** | **Trace** |
|---|---|---|---|---|
| >90% | 55 | 36 | 19 | 0 |
| 80% | 4 | 0 | 3 | 1 |
| 70% | 5 | 0 | 3 | 2 |
| 60% | 6 | 0 | 3 | 3 |
| 50% | 10 | 0 | 0 | 10 |
| 40% | 11 | 0 | 1 | 10 |
| 30% | 12 | 0 | 1 | 11 |
| 20% | 20 | 0 | 0 | 20 |
| 10% | 40 | 0 | 0 | 40 |
| SUM | 163 | 36 | 30 | 97 |

(B)

| *f* | **Total** | **Major** | **Minor** | **Trace** |
|---|---|---|---|---|
| >90% | 64 | 48 | 16 | 0 |
| 80% | 13 | 1 | 12 | 0 |
| 70% | 11 | 0 | 11 | 0 |
| 60% | 17 | 0 | 17 | 0 |
| 50% | 30 | 1 | 23 | 6 |
| 40% | 40 | 0 | 17 | 23 |
| 30% | 42 | 0 | 4 | 38 |
| 20% | 81 | 0 | 1 | 80 |
| 10% | 165 | 0 | 1 | 164 |
| SUM | 463 | 50 | 102 | 311 |

**Table S3.** The composition, frequency, and the class of N-glycans assigned (A) healthy control and (B) patient group.

(A)

| Composition | Frequency | Class |
|---|---|---|
| 3_2_0_0_0 | 100.0% | MAJOR |
| 3_2_1_0_0 | 100.0% | MAJOR |
| 5_4_1_2_0 | 100.0% | MAJOR |
| 6_5_0_1_0 | 100.0% | MAJOR |
| 4_3_0_1_0 | 100.0% | MAJOR |
| 6_4_1_1_0 | 100.0% | MAJOR |
| 4_3_0_0_0 | 100.0% | MAJOR |
| 3_3_1_0_0 | 100.0% | MAJOR |
| 6_4_0_1_0 | 100.0% | MAJOR |
| 5_4_1_1_0 | 100.0% | MAJOR |
| 4_3_1_0_0 | 100.0% | MAJOR |
| 3_4_1_0_0 | 100.0% | MAJOR |
| 5_4_0_0_1 | 100.0% | MAJOR |
| 7_4_0_1_0 | 100.0% | MAJOR |
| 3_4_0_0_0 | 100.0% | MAJOR |
| 5_4_1_0_0 | 100.0% | MAJOR |
| 5_4_0_0_0 | 100.0% | MAJOR |
| 5_4_0_1_0 | 100.0% | MAJOR |
| 8_2_0_0_0 | 100.0% | MAJOR |
| 9_2_0_0_0 | 100.0% | MAJOR |
| 5_2_0_0_0 | 100.0% | MAJOR |
| 5_4_0_2_0 | 100.0% | MAJOR |
| 3_3_0_0_0 | 100.0% | MAJOR |
| 4_4_1_0_0 | 100.0% | MAJOR |
| 7_2_0_0_0 | 100.0% | MAJOR |
| 6_2_0_0_0 | 100.0% | MAJOR |
| 4_2_0_0_0 | 100.0% | MAJOR |
| 5_4_0_1_1 | 100.0% | MAJOR |
| 4_4_0_1_0 | 100.0% | MAJOR |
| 4_4_0_0_0 | 100.0% | MAJOR |
| 6_4_0_2_0 | 100.0% | MAJOR |
| 6_3_0_1_0 | 100.0% | MAJOR |
| 6_4_0_1_1 | 100.0% | MAJOR |
| 5_3_0_1_0 | 100.0% | MAJOR |
| 5_3_1_0_0 | 100.0% | MAJOR |
| 5_5_2_0_0 | 92.7% | MAJOR |
| 5_3_0_0_0 | 100.0% | MINOR |
| 6_3_1_0_0 | 100.0% | MINOR |
| 6_3_1_0_1 | 100.0% | MINOR |
| 5_4_0_0_2 | 100.0% | MINOR |
| 4_4_1_1_0 | 100.0% | MINOR |
| 6_4_1_0_0 | 100.0% | MINOR |
| 4_3_1_1_0 | 100.0% | MINOR |
| 4_3_0_0_1 | 100.0% | MINOR |
| 6_3_0_0_0 | 100.0% | MINOR |
| 6_5_0_2_0 | 100.0% | MINOR |
| 6_4_0_0_1 | 97.6% | MINOR |

| | | |
|---|---|---|
| 7_4_1_0_0 | 97.6% | MINOR |
| 7_4_0_0_0 | 95.1% | MINOR |
| 10_2_0_0_0 | 92.7% | MINOR |
| 7_6_1_0_1 | 92.7% | MINOR |
| 5_3_1_1_0 | 92.7% | MINOR |
| 6_4_0_0_0 | 92.7% | MINOR |
| 6_3_1_1_0 | 90.2% | MINOR |
| 4_4_0_0_1 | 90.2% | MINOR |
| 4_3_2_0_0 | 85.4% | MINOR |
| 6_5_2_0_0 | 82.9% | MINOR |
| 6_3_0_0_1 | 80.5% | MINOR |
| 5_4_2_0_0 | 78.0% | MINOR |
| 6_4_3_0_0 | 78.0% | MINOR |
| 6_5_0_3_0 | 75.6% | MINOR |
| 3_4_0_0_2 | 65.9% | MINOR |
| 6_5_3_2_1 | 65.9% | MINOR |
| 4_5_0_0_2 | 61.0% | MINOR |
| 7_6_0_0_0 | 46.3% | MINOR |
| 4_4_3_0_1 | 39.0% | MINOR |

(B)

| Composition | Frequency | Class |
|---|---|---|
| 3_2_0_0_0 | 100.0% | MAJOR |
| 3_2_1_0_0 | 100.0% | MAJOR |
| 3_4_3_0_0 | 100.0% | MAJOR |
| 4_4_3_0_0 | 100.0% | MAJOR |
| 5_4_1_2_0 | 100.0% | MAJOR |
| 4_3_0_1_0 | 100.0% | MAJOR |
| 6_4_1_1_0 | 100.0% | MAJOR |
| 4_3_0_0_0 | 100.0% | MAJOR |
| 7_4_0_0_0 | 100.0% | MAJOR |
| 3_3_1_0_0 | 100.0% | MAJOR |
| 6_4_0_1_0 | 100.0% | MAJOR |
| 5_3_0_0_0 | 100.0% | MAJOR |
| 5_4_1_1_0 | 100.0% | MAJOR |
| 6_3_1_0_0 | 100.0% | MAJOR |
| 4_3_1_0_0 | 100.0% | MAJOR |
| 3_4_1_0_0 | 100.0% | MAJOR |
| 5_4_0_0_1 | 100.0% | MAJOR |
| 7_4_0_1_0 | 100.0% | MAJOR |
| 6_4_0_0_1 | 100.0% | MAJOR |
| 3_4_0_0_0 | 100.0% | MAJOR |
| 5_4_1_0_0 | 100.0% | MAJOR |
| 5_4_0_0_0 | 100.0% | MAJOR |
| 5_4_0_1_0 | 100.0% | MAJOR |
| 8_2_0_0_0 | 100.0% | MAJOR |
| 9_2_0_0_0 | 100.0% | MAJOR |
| 5_4_0_0_2 | 100.0% | MAJOR |
| 5_2_0_0_0 | 100.0% | MAJOR |
| 5_4_0_2_0 | 100.0% | MAJOR |
| 3_3_0_0_0 | 100.0% | MAJOR |

| | | |
|---|---|---|
| 4_4_1_0_0 | 100.0% | MAJOR |
| 7_2_0_0_0 | 100.0% | MAJOR |
| 6_2_0_0_0 | 100.0% | MAJOR |
| 4_2_0_0_0 | 100.0% | MAJOR |
| 4_4_1_1_0 | 100.0% | MAJOR |
| 5_4_0_1_1 | 100.0% | MAJOR |
| 4_4_0_1_0 | 100.0% | MAJOR |
| 4_4_0_0_0 | 100.0% | MAJOR |
| 6_4_0_2_0 | 100.0% | MAJOR |
| 6_4_1_0_0 | 100.0% | MAJOR |
| 6_3_0_1_0 | 100.0% | MAJOR |
| 5_3_0_1_0 | 100.0% | MAJOR |
| 5_3_1_0_0 | 100.0% | MAJOR |
| 4_4_2_1_0 | 98.9% | MAJOR |
| 10_2_0_0_0 | 98.9% | MAJOR |
| 6_3_1_0_1 | 98.9% | MAJOR |
| 7_4_1_0_0 | 98.9% | MAJOR |
| 5_5_2_0_0 | 98.9% | MAJOR |
| 6_4_0_1_1 | 97.8% | MAJOR |
| 3_4_0_0_2 | 81.5% | MAJOR |
| 4_4_3_0_1 | 54.3% | MAJOR |
| 5_4_3_0_0 | 100.0% | MINOR |
| 6_3_0_0_0 | 100.0% | MINOR |
| 6_3_1_1_0 | 100.0% | MINOR |
| 5_3_1_1_0 | 100.0% | MINOR |
| 4_4_0_0_1 | 100.0% | MINOR |
| 7_7_5_2_2 | 97.8% | MINOR |
| 6_5_0_2_0 | 97.8% | MINOR |
| 4_3_1_1_0 | 96.7% | MINOR |
| 7_4_2_0_0 | 96.7% | MINOR |
| 6_5_1_0_0 | 96.7% | MINOR |
| 6_4_0_0_0 | 96.7% | MINOR |
| 4_5_2_0_0 | 95.7% | MINOR |
| 4_3_0_0_1 | 94.6% | MINOR |
| 6_5_0_0_0 | 94.6% | MINOR |
| 7_7_3_2_1 | 93.5% | MINOR |
| 6_4_2_0_0 | 91.3% | MINOR |
| 6_7_4_0_0 | 89.1% | MINOR |
| 6_3_0_0_1 | 89.1% | MINOR |
| 6_5_0_1_0 | 88.0% | MINOR |
| 6_5_1_1_0 | 88.0% | MINOR |
| 6_5_0_0_1 | 88.0% | MINOR |
| 3_6_0_0_1 | 87.0% | MINOR |
| 7_6_3_2_1 | 84.8% | MINOR |
| 6_3_2_0_0 | 81.5% | MINOR |
| 6_7_4_1_0 | 80.4% | MINOR |
| 4_3_0_0_2 | 80.4% | MINOR |
| 6_5_0_3_0 | 80.4% | MINOR |
| 11_2_0_0_0 | 80.4% | MINOR |
| 5_5_1_0_0 | 79.3% | MINOR |
| 4_7_3_1_0 | 78.3% | MINOR |
| 5_7_3_1_0 | 77.2% | MINOR |

| | | |
|---|---|---|
| 5_7_4_1_0 | 77.2% | MINOR |
| 7_4_1_1_0 | 77.2% | MINOR |
| 4_5_1_0_0 | 76.1% | MINOR |
| 4_3_2_0_0 | 76.1% | MINOR |
| 6_4_3_0_0 | 72.8% | MINOR |
| 7_4_1_0_1 | 71.7% | MINOR |
| 7_7_3_4_0 | 70.7% | MINOR |
| 5_4_2_0_0 | 70.7% | MINOR |
| 4_7_4_1_0 | 68.5% | MINOR |
| 3_6_0_0_4 | 68.5% | MINOR |
| 7_7_1_0_4 | 68.5% | MINOR |
| 7_7_1_3_1 | 68.5% | MINOR |
| 7_6_3_3_0 | 67.4% | MINOR |
| 4_3_2_1_0 | 65.2% | MINOR |
| 4_3_2_0_2 | 65.2% | MINOR |
| 6_7_2_2_0 | 64.1% | MINOR |
| 7_7_2_3_1 | 64.1% | MINOR |
| 4_6_1_1_0 | 64.1% | MINOR |
| 4_7_0_1_0 | 64.1% | MINOR |
| 5_6_5_2_0 | 63.0% | MINOR |
| 7_7_5_1_2 | 62.0% | MINOR |
| 7_7_5_0_3 | 62.0% | MINOR |
| 3_3_2_0_0 | 62.0% | MINOR |
| 6_6_2_0_0 | 62.0% | MINOR |
| 5_6_4_1_0 | 60.9% | MINOR |
| 4_3_1_0_2 | 59.8% | MINOR |
| 4_4_2_0_0 | 59.8% | MINOR |
| 6_7_3_2_0 | 58.7% | MINOR |
| 5_7_2_1_0 | 58.7% | MINOR |
| 4_5_2_1_0 | 58.7% | MINOR |
| 7_7_5_0_4 | 57.6% | MINOR |
| 7_7_1_2_2 | 57.6% | MINOR |
| 7_6_1_0_1 | 55.4% | MINOR |
| 7_7_5_0_1 | 55.4% | MINOR |
| 5_6_0_1_0 | 55.4% | MINOR |
| 6_3_2_1_0 | 55.4% | MINOR |
| 6_4_2_1_0 | 55.4% | MINOR |
| 7_4_0_0_3 | 54.3% | MINOR |
| 4_5_0_0_2 | 54.3% | MINOR |
| 6_6_5_3_0 | 53.3% | MINOR |
| 6_5_2_2_0 | 53.3% | MINOR |
| 5_4_3_0_3 | 53.3% | MINOR |
| 5_7_4_0_0 | 52.2% | MINOR |
| 4_6_1_0_3 | 52.2% | MINOR |
| 5_7_0_0_1 | 52.2% | MINOR |
| 5_4_3_1_0 | 51.1% | MINOR |
| 7_7_1_4_0 | 50.0% | MINOR |
| 7_7_3_1_0 | 50.0% | MINOR |
| 7_6_1_3_1 | 48.9% | MINOR |
| 5_3_2_1_0 | 48.9% | MINOR |
| 3_4_2_0_0 | 48.9% | MINOR |
| 5_5_4_2_0 | 47.8% | MINOR |

| | | |
|---|---|---|
| 6_5_2_0_0 | 46.7% | MINOR |
| 4_6_3_0_0 | 46.7% | MINOR |
| 3_6_4_0_0 | 46.7% | MINOR |
| 6_4_0_0_3 | 45.7% | MINOR |
| 7_7_3_2_2 | 45.7% | MINOR |
| 7_7_3_0_1 | 43.5% | MINOR |
| 7_6_0_0_0 | 43.5% | MINOR |
| 7_4_3_0_0 | 42.4% | MINOR |
| 5_7_4_2_0 | 41.3% | MINOR |
| 5_6_5_0_0 | 41.3% | MINOR |
| 7_7_3_1_2 | 41.3% | MINOR |
| 6_7_3_1_0 | 41.3% | MINOR |
| 7_7_5_1_3 | 40.2% | MINOR |
| 6_7_5_3_0 | 39.1% | MINOR |
| 6_7_5_0_0 | 39.1% | MINOR |
| 7_7_5_3_1 | 33.7% | MINOR |
| 6_5_3_2_1 | 30.4% | MINOR |
| 4_6_5_1_0 | 26.1% | MINOR |
| 7_7_1_0_2 | 10.9% | MINOR |

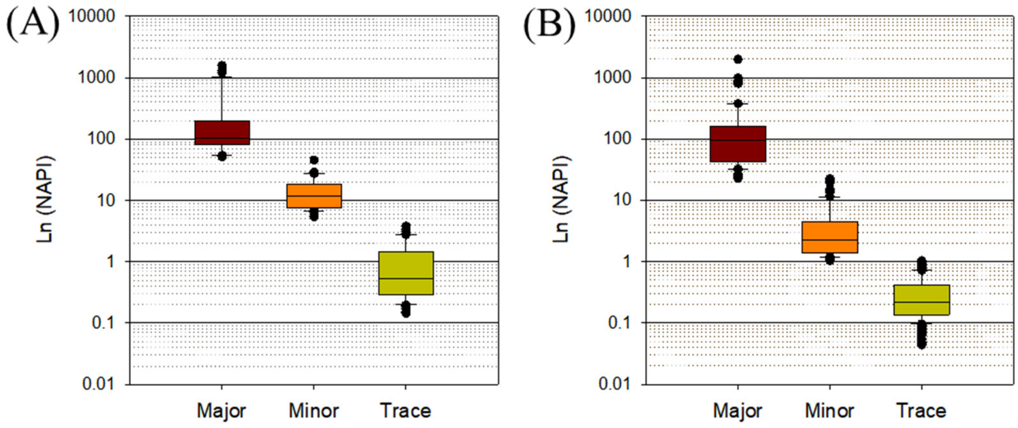


**Figure S1.** The comparison of peak intensity between Peak Major class, Minor class, and Trace groups. (**A**) healthy control group, (**B**) Osteoarthritis patient group.

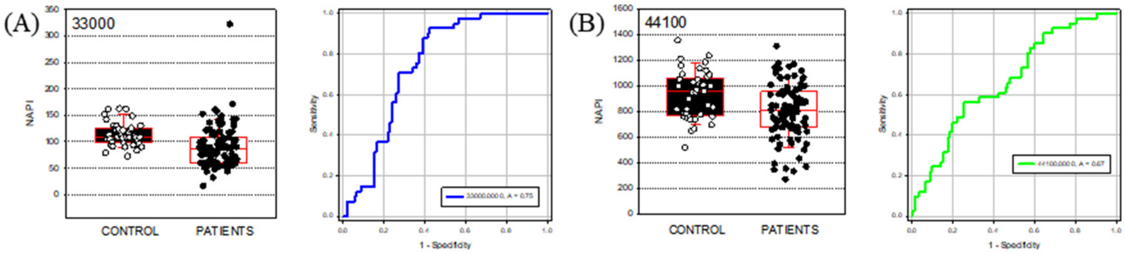


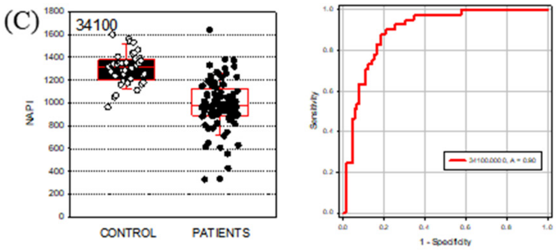


| m/z | Note | Control | | | Patient | | | *p*-value | AUC | P/C ratio |
|---|---|---|---|---|---|---|---|---|---|---|
| | | AVE | SD | CV | AVE | SD | CV | | | |
| 1113.4071 | 33000 | 114 | ± 22 | 19.4% | 91 | ± 41 | 44.7% | **8.7.E-04** | **0.75** | -1.3 |
| 1624.5972 | 44100 | 932 | ± 185 | 19.9% | 800 | ± 215 | 26.9% | **9.5.E-04** | **0.67** | -1.2 |
| 1462.5444 | 34100 | 1302 | ± 138 | 10.6% | 985 | ± 220 | 22.3% | **3.5.E-14** | **0.90** | -1.3 |

**Figure S2.** Example of biomarkers with high AUC but low P/C ratio. (**A**) and (**B**) has high *p*-value and low AUC. (**C**) has low *p*-value and high AUC but a similar P/C ratio with (**A**) and (**B**). Although N-glycan (**C**) has a high AUC of 0.90 values of OA patients and healthy control made clusters in close range.
